# Economic analysis of palonosetron versus granisetron in the standard triplet regimen for preventing chemotherapy-induced nausea and vomiting in patients receiving highly emetogenic chemotherapy in Japan (TRIPLE phase III trial)

**DOI:** 10.1186/s40780-018-0128-9

**Published:** 2018-12-10

**Authors:** Hisanori Shimizu, Kenichi Suzuki, Takeshi Uchikura, Daiki Tsuji, Takeharu Yamanaka, Hironobu Hashimoto, Koichi Goto, Reiko Matsui, Nobuhiko Seki, Toshikazu Shimada, Shunya Ikeda, Naoki Ikegami, Toshihiro Hama, Nobuyuki Yamamoto, Tadanori Sasaki

**Affiliations:** 1 Department of Hospital Pharmaceutics, School of Pharmacy, Showa University, Tokyo, Japan; 2 Department of Pharmacy, Japanese Foundation for Cancer Research, Cancer Institute Hospital, Tokyo, Japan; 3 Department of Clinical Pharmacology & Genetics, School of Pharmaceutical Sciences, University of Shizuoka, Shizuoka, Japan; 4 Department of Biostatistics, Yokohama City University School of Medicine, Yokohama, Japan; 5 Division of Pharmacy, National Cancer Center Hospital, Tokyo, Japan; 6 Department of Thoracic Oncology, National Cancer Center Hospital East, Kashiwa, Japan; 7 Departments of Pharmacy, National Cancer Center Hospital East, Kashiwa, Japan; 8 Division of Medical Oncology, Department of Internal Medicine, Teikyo University School of Medicine, Tokyo, Japan; 9 Center for Clinical Reseach&Trial, Teikyo University Hospital, Tokyo, Japan; 10 Department of Public Health, School of Medicine, International University of Health and Welfare, Chiba, Japan; 11 Graduate School of Public Health, St Lukes International University, Tokyo, Japan; 12 Third Department of Internal Medicine, Wakayama Medical University, Wakayama, Japan; 13 Department of Pharmacy Services, Showa University Hospital, 1-5-8, Hatanodai, Shinagawa-ku, Tokyo, 142-8666 Japan

**Keywords:** Highly emetogenic chemotherapy, 5-HT_3_ receptor antagonist, Palonosetron, Granisetron, Chemotherapy-induced nausea and vomiting, Cost-effectiveness, Real-world data analysis

## Abstract

**Background:**

We conducted an economic assessment using test data from the phase III TRIPLE study, which examined the efficacy of a 5-hydroxytryptamine 3 receptor antagonist as part of a standard triplet antiemetic regimen including aprepitant and dexamethasone in preventing chemotherapy-induced nausea and vomiting in patients receiving cisplatin-based highly emetogenic chemotherapy (HEC).

**Methods:**

We retrospectively investigated all medicines prescribed for antiemetic purposes within 120 h after the initiation of cisplatin administration during hospitalization. In the TRIPLE study, patients were assigned to treatment with granisetron (GRA) 1 mg (*n* = 413) or palonosetron (PALO) 0.75 mg (*n* = 414). The evaluation measure was the cost-effectiveness ratio (CER) assessed as the cost per complete response (CR; no vomiting/retching and no rescue medication). The analysis was conducted from the public healthcare payer’s perspective.

**Results:**

The CR rates were 59.1% in the GRA group and 65.7% in the PALO group (*P* = 0.0539), and the total frequencies of rescue medication use for these groups were 717 (153/413 patients) and 573 (123/414 patients), respectively. In both groups, drugs with antidopaminergic effects were chosen as rescue medication in 86% of patients. The costs of including GRA and PALO in the standard triplet antiemetic regimen were 15,342.8 and 27,863.8 Japanese yen (JPY), respectively. In addition, the total costs of rescue medication use were 73,883.8 (range, 71,106.4–79,017.1) JPY for the GRA group and 59,292.7 (range, 57,707.5–60,972.8) JPY for the PALO group. The CERs (JPY/CR) were 26,263.4 and 42,628.6 for the GRA and PALO groups, respectively, and the incremental cost-effectiveness ratio (ICER) between the groups was 189,171.6 (189,044.8–189,215.5) JPY/CR.

**Conclusions:**

We found that PALO was more expensive than GRA in patients who received a cisplatin-based HEC regimen.

**Electronic supplementary material:**

The online version of this article (10.1186/s40780-018-0128-9) contains supplementary material, which is available to authorized users.

## Background

Chemotherapy-induced nausea and vomiting (CINV) is an uncomfortable side effect that must be considered in patients with cancer. Prochlorperazine, high-dose metoclopramide, corticosteroids, and 5-hydroxytryptamine 3 (5-HT_3_) receptor antagonists (RAs) were demonstrated to significantly improve CINV symptoms within 24 h after the initiation of chemotherapy. Conversely, some patients experience CINV in the delayed phase [1].

Dopamine, serotonin, and substance P act as neurotransmitters for chemoreceptors. Aprepitant (APR), a neurokinin-1 (NK-1) RA, was developed in the 2000s (international birth date, March 2003) [2, 3]. Several reports discussed the cost-effectiveness of NK-1 RAs [4–8]. Meanwhile, current antiemetic guidelines recommend a three-drug combination consisting of a 5-HT_3_ RA, dexamethasone (DEX), and APR for patients receiving highly emetogenic chemotherapy (HEC) [9–11]. Palonosetron (PALO), a second-generation 5-HT_3_ RA with high binding affinity for the serotonin receptor that is expected to have antiemetic efficacy against delayed-phase CINV, reached the market during the same period as APR (international birth date, July 25, 2003) [12–14]. The PROTECT study conducted in Japan [15] verified the superiority of PALO, demonstrating that a regimen including a novel 5-HT_3_ RA, PALO, and DEX is better than the standard regimen of GRA and DEX in preventing CINV during the delayed phase in patients with cancer receiving HEC. However, APR could not be considered in this study because it had not been approved in Japan.

A randomized double-blind controlled trial (TRIPLE study [16]) was conducted to verify the prophylactic antiemetic effect of APR as part of a combination antiemetic regimen expected to be effective during the delayed phase of CINV. The primary endpoint of the TRIPLE study was a complete response (CR; no vomiting/retching and no rescue medication) within 120 h after the initiation of cisplatin treatment. Eligible patients were randomly allocated to double-blind treatment with either GRA (1 mg) or PALO (0.75 mg) as part of triplet antiemetic regimens for cisplatin-based HEC.

Although the primary endpoint was not met and the superiority of PALO was not demonstrated in this clinical trial (*P* = 0.0539), PALO displayed efficacy superior to GRA in terms of controlling CINV, especially in the delayed phase.

No previous study directly compared the cost-effectiveness of GRA and PALO, both in combination with APR and DEX, in preventing CINV in patients treated with cisplatin-based chemotherapy in terms of the detailed cost of antiemetics including rescue medication. As price information is available for all drugs covered by Japan’s health insurance program, it is possible to clarify the drug costs of antiemetics used by various patient groups and the cost-effectiveness ratio (CER) per instance of vomiting suppression as economic evidence. This research sought to clarify the cost-effectiveness of the triple antiemetic combinations and conduct real-world cost analysis using data from the TRIPLE study.

## Methods

### Patients

In total, 827 patients receiving cisplatin-based HEC enrolled in a randomized, double-blind, multicenter phase III trial to validate the superiority of PALO (0.75 mg) over GRA (1 mg) were evaluated. The study design details and primary results of our phase III trial were described previously [16].

This study was conducted in accordance with the Japanese Ethical Guidelines for Medical and Health Research Involving Human Subjects. The personal information of all subjects was deleted, and anonymized clinical data were analyzed retrospectively. In addition to the economic analysis of data from our phase III trial, we investigated and analyzed medical expenses based on data for receipt information during hospitalization in the Cancer Institute Hospital of the Japanese Foundation for Cancer Research after receiving approval from the clinical research ethics review committee.

### Treatment

Patients with cisplatin-naive solid tumors were eligible for this economic analysis if they were scheduled to start their first cycle of chemotherapy including a cisplatin dose ≥50 mg/m^2^ upon hospital admission. Patients with previous cisplatin use could be enrolled if they received the drug > 3 months before enrollment. The inclusion and exclusion criteria were described previously [16].

All patients received GRA or PALO combined with APR and DEX. The standard prophylactic antiemetic regimen to be used during the first cycle of HEC consisted of intravenous PALO (0.75 mg) or GRA (1 mg) on day 1, in addition to oral APR (125 mg on day 1 and 80 mg/day on days 2–3) and intravenous DEX phosphate sodium (12 mg [equivalent to 9.9 mg of DEX] on day 1 and 8 mg/day [equivalent to 6.6 mg of DEX] on days 2–4). On day 1, patients received PALO or GRA together with DEX as an infusion over < 15 min starting at least 30 min before cisplatin was administered. Patients received APR at least 60 min before cisplatin was administered on day 1 and before breakfast on days 2–3.

### Analysis method

#### Measurement of the effects of antiemetic therapy on nausea and vomiting

The CR rates in the TRIPLE study were 59.1% (244/413 patients) for the GRA group and 65.7% (272/414 patients) for the PALO group.

Regarding the case of CR or non-CR, we devised three categories according to the development of overall CINV as well as acute and delayed CINV (i.e.,., category 1, CR [0–120 h]; category 2, non-CR delayed; category 3, non-CR acute) and then retrospectively investigated the direct medical costs of antiemetics, the number of patients with vomiting, and the number of nausea episodes during the observation periods (Additional file 1: Fig. S1).

### Drug cost of antiemetics

The cost of antiemetic drugs was calculated on the basis of the National Health Insurance drug price standard in 2012, during which the TRIPLE study was conducted, and considered the direct costs of medical care. Because generic alternatives were adopted in several institutions, we calculated the cost of treatment using the prices of both branded and generic drugs if the indication of both medicines was the same.

### Study pharmacists and case report form (CRF)

All patients evaluated in this study were hospitalized during the 5-day period of observation. This minimized the effect of external factors on the onset of nausea and vomiting, such as carsickness or smell, for patients receiving chemotherapy. In the study, study pharmacists at each center who were blinded to treatment allocation evaluated the efficacy endpoints for each patient daily using diary and interview data to ensure a rigorous assessment of nausea and vomiting and rescue medication use. In this analysis, additional antiemetic usage (i.e., rescue medication) was investigated by collecting the actual usage data listed in the CRF of the TRIPLE study.

Regarding rescue medication, the intravenous solution was unified as 50 mL of normal saline to dissolve the rescue medication when an injection preparation was chosen, and the cost of the infusion was also added to the medical cost. The use of generic drugs was noted. In addition, in cases in which multiple dosage forms were distributed but dosage forms were not listed, they were recorded as the injection form and branded drug.

### Calculation of the cost-effectiveness ratio (CER)

The mean cost per patient was calculated from the true cost of antiemetics used in each group. The CER was obtained by dividing the mean cost by the number of CRs. Meanwhile, the incremental cost-effectiveness ratio (ICER) was calculated as the difference in mean cost between the groups divided by the difference in CR rates between the groups. In addition, the ICER range was calculated via a one-way sensitivity analysis of branded and generic drugs as rescue medication.

### Analytical viewpoint and sensitivity analysis

The cost-effectiveness analysis was performed from the public healthcare payer’s perspective. The uncertainty of the results was explored via sensitivity analysis of uncertain factors. Because the adoption of antiemetics (i.e.,., branded or generic) differed among the institutions, we conducted one-way sensitivity analysis to calculate the drug cost for generic or branded drug use.

### Cost of hospitalization according to the duration of treatment

To calculate the total medical cost of hospitalization related to chemotherapy including cisplatin and to clarify the actual cost of antiemetic medication, hospitalization expenses for 59 patients enrolled in the TRIPLE study at the Cancer Institute Hospital, Japanese Foundation for Cancer Research, were extracted and converted into a performance-based payment format using medical accounting cost data from the Diagnosis Procedure Combination database. Costs were investigated for both the total medical cost and the cost of drugs.

## Results

### Patient characteristics

In total, 827 patients (414 in the PALO group and 413 in the GRA group) were evaluated for efficacy at 20 Japanese institutions between July 2011 and June 2012 in the TRIPLE study, and all patients were included in this economic analysis. The baseline characteristics of patients in each treatment group are summarized in Table 1. All baseline demographic parameters were similar between the groups.

**Table 1:** Patients Characteristics

	Gra (*n* = 413)	Palo (*n* = 414)
Age, ≥60 years	290 (70.2%)	291 (70.3%)
median (range)	64 (25–83)	63 (31–77)
Gender, male	309 (74.8%)	307 (74.2%)
ECOG Performance Status		
0	282 (68.3%)	276 (66.7%)
1	126 (30.5%)	136 (32.9%)
2	5 (1.2%)	2 (0.5%)
Primary tumor site		
Lung	265 (64.2%)	245 (59.2%)
Esophageal	58 (14.0%)	56 (13.5%)
Gastric	51 (12.4%)	65 (15.7%)
Head and neck	23 (5.6%)	26 (6.3%)
Other	16 (3.9%)	22 (5.3%)
CDDP dose administered (mg/m^2^)		
< 60	67 (16.2%)	65 (15.7%)
≥ 60, < 70	48 (11.6%)	57 (13.8%)
≥ 70, < 80	243 (58.8%)	227 (54.8%)
≥ 80	55 (13.3%)	65 (15.7%)
Prior chemotherapy with platinum more than 3 months earlier	21 (5.1%)	16 (3.9%)

A total of 842 patients were enrolled at 20 Japanese centers between July 2011 and June 2012 and randomly assigned to either Arm PALO or GRA. 14 patients were excluded from analyses, leaving 828 patients evaluable for safety (safety population). One patient in Arm PALO had no efficacy data due to a serious adverse event soon after the antiemetic treatment. Thus, the FAS comprised 827 patients (414 in Arm PALO and 413 in Arm GRA)

### Drug cost of antiemetics

One tablet of lorazepam 0.5 mg, which was the least expensive generic benzodiazepine, cost only 5 Japanese yen (JPY), whereas one ampoule of palonosetron hydrochloride injection 0.75 mg (branded medicine), the most expensive medicine, cost 14,522 JPY. The second most expensive medicine was a tri-pack of APR (branded medicine, 11,244.8 JPY). The costs of standard prophylactic antiemetic treatment were 15,342.8 JPY for the GRA group and 27,863.8 JPY for the PALO group, producing a difference of 12,521 JPY. The prices of drugs used as antiemetics to prevent CINV are presented in Additional file 2: Table S1.

In addition, the medical cost of antiemetics based on the revision of the National Health Insurance drug price standard in 2016 is presented in Additional file 3: Table S2 (rate: 1 US dollar = 110.57 JPY, 1 euro = 128.85 JPY, July. 4, 2018).

### Effectiveness and incidence of CINV

CR rates during the 120-h period after the initiation of the first cycle of cisplatin treatment were 59.1% in the GRA group and 65.7% in the PALO group (*P* = 0.0539).

Vomiting within 120 h after cisplatin administration during hospitalization occurred in 75 patients (18.2%) in the GRA group and 65 patients (15.7%) in the PALO group. The total frequencies of nausea during the observation period were 1092 and 887 in the GRA and PALO groups, respectively. Vomiting, nausea, and rescue medication use were less frequent in the PALO group, whereas the CINV-preventive effect was superior for GRA (Table 2).

**Table 2:** Frequency of rescue medication and Cost-Effectiveness Ratio

		Rescue medication				Vomiting	Nausea			Cost of prophylaxis regimen (JPY)	Costs of the antiemetics (JPY)	CR (Overall)	CER (JPY/CR)
		Frequency (*n*)	Mean cost (SD)	Amount total (JPY)	Sensitivity analysis	Pts. No. (%)	Frequency	Median (range)	Mode				
GRA (1 mg)	total (*n* = 413)	717 (*n* = 153)	178.9 (712.7)	73,883.8	71,106.4 - 79,017.1	75 (18.2%)	1092	1 (0–15)	0	15,342.8	15,521.7	0.591	26,263.4
	category 1 (*n* = 244)	0	0	0	–	0	199	0 (0–10)	0				
	category 2 (*n* = 135)	468 (*n* = 122)	382.7 (1078)	51,663.5	49,709.9 - 54,279.8	52 (38.5%)	631	5 (0–15)	5				
	category 3 (*n* = 34)	249 (*n* = 31)	635.5 (991.9)	22,220.3	21,396.5 - 24,737.3	23 (67.6%)	262	8 (0–15)	5				
PALO (0.75 mg)	total (*n* = 414)	573 (*n* = 123)	143.2 (795.8)	59,292.7	57,707.5 - 60,972.8	65 (15.7%)	887	1 (0–13)	0	27,863.8	28,007	0.657	42,628.6
	category 1 (*n* = 272)	0	0	0	–	0	195	0 (0–7)	0				
	category 2 (*n* = 108)	356 (*n* = 92)	286.5 (1003.3)	30,942.6	30,007.6 - 32,242.3	44 (40.7%)	485	4 (0–13)	3				
	category 3 (*n* = 34)	217 (*n* = 31)	833.8 (1983.3)	28,350.1	27,699.9 - 28,730.5	21 (61.8%)	207	6.5 (0–13)	7				

With regard to a case of CR or non-CR, we set three categories according to the development of CINV in overall, acute and delayed phase (i.e. category 1: CR (0–120 h), category 2: non-CR delayed, category 3: non-CR acute) and then investigated retrospectively the direct medical costs of antiemetics, the number of patients with vomiting, and the number of nausea episodes during observation periods

range = minimum to maximum

CR: Complete Response (no vomiting/retching and no rescue medication)

CER: Cost-Effectiveness Ratio

In addition, the proportion of patients with vomiting was higher in the acute phase (category 3) of non-CR based on the CR judgment than in the delayed phase (category 2).

### Rescue medication

The total frequencies of rescue medication use within 120 h after cisplatin administration during hospitalization were 717 (153/413 patients) in the GRA group and 573 (123/414 patients) in the PALO group. The total additional costs for rescue medication use were 73,883.8 and 59,292.7 JPY in the GRA and PALO groups, respectively.

In addition, the ranges of additional expenses calculated by one-way sensitivity analysis to determine drug costs in cases of generic (minimum) or branded (maximum) drugs were 71,106.4–79,017.1 JPY for the GRA group and 57,707.5–60,972.8 JPY for the PALO group (Table 2). The mean additional expense of rescue medication use was higher in category 3 than in category 2.

### Antiemetics selected as rescue medication

**Fig. 1:**
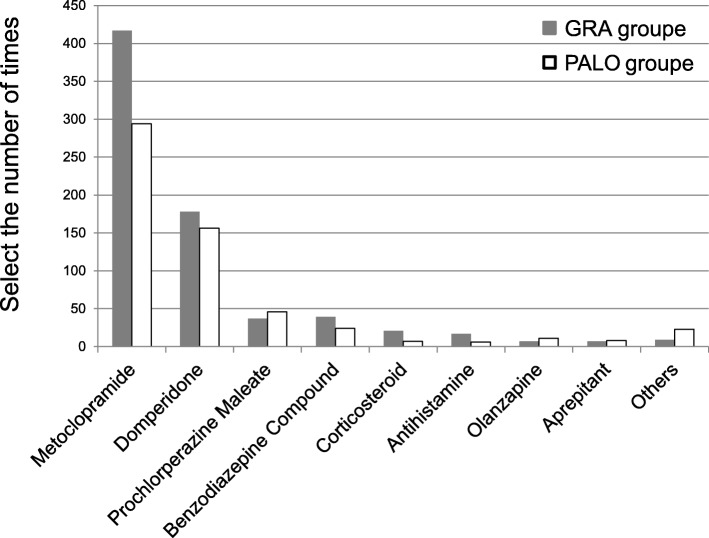
Antiemetics selected as rescue medication. The total frequencies of rescue medication use within 120 h after cisplatin administration during hospitalization were 717 (153/413 patients) in the granisetron (GRA) group and 573 (123/414 patients) in the palonosetron (PALO) group. Antidopaminergic agents (metoclopramide, domperidone, and prochlorperazine maleate) were used as rescue medication for CINV in 86% of patients in each treatment group

Antidopaminergic agents (metoclopramide, domperidone, and prochlorperazine maleate) were selected as medication for CINV for 86% of patients in each treatment group (Fig. 1).

The usage of other medicines (i.e., benzodiazepine compound, corticosteroids, antihistamines) was also similar between the groups. Olanzapine was used 7 (0.96%) and 11 times (1.91%) in the GRA and PALO groups, respectively.

### The cost-effectiveness ratio

The CERs for the GRA and PALO groups were 26,263.4 and 42,628.6 JPY/CR, respectively (Table 2). The ICER required to achieve the observed difference in overall phase CR rates between the two treatment groups of 6.6% in favor of the PALO group was 189,171.6 JPY/CR. The range of the ICER estimated via our one-way sensitivity analysis was 189,044.8–189,215.5 JPY/CR.

### Cost of hospitalization over duration of treatment

We also conducted medical expenses analysis based on the data for receipt information during hospitalization in the Cancer Institute Hospital of the Japanese Foundation for Cancer Research. The cost data before 2011 on this facility had become unavailable by refurbishing the medical computer system, and accordingly among 59 patients enrolled in the TRIPLE study were used the data of 43 patients. The results of our retrospective survey are shown in Table 3.

**Table 3:** Cost of hospitalization in the duration of treatment

		Total	GRA	PALO
		*n* = 43	*n* = 23	*n* = 20
Total medical cost (JPY)	mean	1,113,138.8	1,199,584.35	1,013,726.5
	SD	870,137.9	1,043,255.9	627,690.9
	range	371,080 - 4,801,680	371,080 - 4,801,680	529,060 - 2,839,830
Drug costs (JPY)	mean	195,716.7	227,712.6	158,921.5
	SD	152,411.2	189,732.7	83,676.9
	range	61,060 - 848,610	70,800 - 848,610	61,060 - 449,030
Regimen	CDDP + GEM	13	6	7
	CDDP + PEM	5	4	1
	CDDP + VP16	8	3	5
	CDDP	5	3	2
	CDDP + CPT	4	3	1
	CDDP + DTX	6	3	3
	CDDP + S1	2	1	1
Tumour type	NSCLC	25	12	13
	SCLC	12	6	6
	Cervical esophagus caner	1	1	0
	Pharyngeal caner	4	3	1
	Nasal caner	1	1	0

Among 59 patients enrolled in TRIPLE study from the Cancer Institute Hospital, Japanese Foundation for Cancer Research, 43 patients were enabled to obtain the receipt information of hospitalization

The cost data before 2011 on this facility had become unavailable by refurbishing the medical computer system, and accordingly among 59 patients enrolled in the TRIPLE study were used the data of 43 patients

range = minimum to maximum

CDDP: cisplatin, GEM: gemcitabine,PEM: pemetrexed, VP16: etoposide, CPT: irinotecan,

DTX: docetaxel, S1: tegafur/gimeracil/oteracil

NSCLC: non-small cell lung cancer, SCLC: small cell lung cancer

The mean medical cost (SD) during hospitalization related to cancer chemotherapy was 1,113,138.8 (870,137.9) JPY, including a drug cost of 195,716.7 (152,411.2) JPY. The total medical costs were 1,199,584.35 (1,043,255.9) JPY in the GRA group (*n* = 23) and 1,013,726.5 (627,690.9) JPY in the PALO group (*n* = 20). In addition, the drug costs in these groups were 227,712.6 (189,732.7) and 158,921.5 (83,676.9) JPY, respectively. Meanwhile, costs related to antiemetic prophylaxis and added rescue medication use were higher in the PALO group.

## Discussion

We conducted this economic analysis to obtain economic evidence without using a simulation model in addition to clinical evidence of the efficacy of standard triplet antiemetic therapy in the TRIPLE study [16].

From this economic evaluation, we determined the CER and ICER, which served as indices of the cost-effectiveness of standard triplet antiemetic therapy for preventing CINV in patients receiving cisplatin-based HEC regimens in Japan. We expect that these indices will lead to the generation of highly transparent evidence for comparing the cost-effectiveness of novel antiemetics developed in the near future. For example, the usefulness of olanzapine for preventing chemotherapy-induced nausea was recently reported [17], and our indices can evaluate whether new standard prophylactic antiemetic regimens using olanzapine have additional effects corresponding to the cost of HEC. Moreover, it appears useful to identify individual drug interactions associated with APR, especially strategies for patients with limited steroid use [18–20] and combinations of inexpensive antiemetics.

Although the TRIPLE study was a randomized controlled trial, the eligibility criteria for the study were relatively similar to clinical practice guidelines, and patient registration in the study was not influenced by life-related prognostic factors that verify the effect of chemotherapy; thus, the results of this economic study approximately could be considered real-world data.

In addition, the protocol of the TRIPLE study stipulated that a branded medicine (granisetron 1 mg) was the standard triplet antiemetic in a prophylactic regimen. Because generic drugs are recommended in clinical practice, it is considered that the ICER will further increase due to increased generic drug use.

The difference in the incremental drug cost per patient and cycle for antiemetic prophylaxis in the two treatment groups was 12,521 JPY in favor of GRA. Conversely, the average additional costs for rescue medication use were 178.9 JPY in the GRA group and 143.2 JPY in the PALO group. The cost difference of the standard triplet prophylaxis regimen was approximately 350 times larger than that of the average additional cost for rescue medication, reflecting the high cost of PALO. Furthermore, the range of the ICER calculated from the one-way sensitivity analysis was 189,044.8–189,215.5 JPY/CR, indicating that the influence of rescue medication use on treatment costs is small. Recently published literature using a simulation model revealed that PALO 0.25 mg was not more cost-effective than GRA 3 mg for patients following HEC in an economic evaluation of a 5-HT_3_ RA and DEX without APR [21]. Our economic study indicated that the difference in price between PALO 0.75 mg and GRA 1 mg was large and that it is the factor most strongly affecting the ICER. Thus, this economic evaluation also revealed that PALO 0.75 mg was more expensive than GRA 1 mg in patients receiving cisplatin-based HEC, in line with a previous report [21].

A national policy is required to suppress soaring medical costs in Japan, and a basic policy to drastically reform the National Health Insurance drug price list has been planned ahead of the introduction of expensive and innovative new medicines [22]. The formal introduction of a cost-effectiveness evaluation is being discussed. In addition, “Guidelines for economic evaluation of healthcare technologies in Japan [23]” have been developed; therefore, there are few reports of economic evaluations in the country. Although one report raised questions regarding pharmacoeconomic evaluations based on randomized controlled trials [24], an urgent need for treatment options based on the efficient use of medical resources and cost-effectiveness exists. Therefore, it is expected that the availability of economic evidence in clinical practice based on real-world cost analyses will be promoted mainly by clinical trial planners, and it is desirable to actively publicize this information [25, 26].

The major limitation of this retrospective research was that it could not track the influence of repeat chemotherapy administration and the additional use of antiemetics to prevent anticipatory CINV, because this study was just focused on the effect of antiemetics within 120 h of the first cycle of cisplatin administration, which is extremely short term. In addition, the other limitation of this research was that this research was a CRF-based analysis limited to the cost of antiemetic drugs during the 5-day observation period. Although it is necessary to analysis excessive cost of prolonged hospitalization to identify better strategy for CINV prevention, we could not conduct such data analysis with available CRF. Therefore, it is possible to confirm the robustness of our results by implementing a simulation model using quality-adjusted life years as a complementary study of cost-effectiveness or conducting a retrospective investigation to clarify the influence of treatment continuation in trial-registered patients on the prognosis of antiemetic therapy.

Antiemetic therapy, a preventive strategy for CINV, should be selected considering for cost-effectiveness and individualization. This retrospective research revealed that GRA was more cost-effective than PALO in patients receiving cisplatin-based HEC regimens. In addition, Tsuji et al. conducted that the risk factor analysis [27] and gene polymorphism research [28] revealed evidence such as predictive factor as a follow-up research of the TRIPLE study. We should verify that predicting patients who should use PALO or patients who can obtain sufficient effect even with combinations of inexpensive antiemetics before treatment of chemotherapy.

Further investigation demonstrated that the minimum and maximum total medical and drug costs differ by more than 10-fold. The high costs were attributed to pemetrexed use (high drug cost) and pharyngeal cancer (high cost of medical examinations). This suggests that the expense of anticancer drugs, which are more expensive than antiemetics, is attracting attention in practical medical care.

## Conclusions

We determined the CER and ICER, which served as indices of the cost-effectiveness of standard triplet antiemetic therapy for preventing CINV in patients receiving cisplatin-based HEC regimens in Japan. Also, we found that PALO 0.75 mg was more expensive than GRA 1 mg in the patients who received the cisplatin-based HEC regimen.

## Additional files

Additional file 1: **Figure S1.** The structure of the category and effect measurement of CINV. The CR rates in the TRIPLE study were 59.1% (244/413 patients) for the GRA group and 65.7% (272/414 patients) for the PALO group. Regarding the case of CR or non-CR, we devised three categories according to the development of overall CINV as well as acute and delayed CINV (category 1, CR [0–120 h]; category 2, non-CR delayed; category 3, non-CR acute). (PPTX 83 kb)

Additional file 2: **Table S1.** Drug cost of antiemetics. (XLS 15 kb)

Additional file 3: **Table S2.** Drug cost of antiemetics on the National Health Insurance drug price standard in 2017. (XLS 18 kb)

## Abbreviations

APR: Aprepitant
CC: Complete Control
CDDP: Cisplatin
CER: Cost-Effectiveness Ratio
CINV: Chemotherapy-Induced Nausea and Vomiting
CR: Complete Response
CRF: Case Report Form
DEX: Dexamethasone
GRA: Granisetron
HEC: highly emetogenic chemotherapy
ICER: Incrimental Cost-Effectiveness Ratio
JPY: Japanease yen
PALO: Palonosetron
QALYs: Quality-adjusted Life years
TC: Total Control
