# Supplementary material for: Economic analysis of palonosetron versus granisetron in the standard triplet regimen for preventing chemotherapy-induced nausea and vomiting in patients receiving highly emetogenic chemotherapy in Japan (TRIPLE phase III trial)
Source: J Pharm Health Care Sci. 2018 Dec 10;4:31. doi: 10.1186/s40780-018-0128-9 (PMC6287343; doi:10.1186/s40780-018-0128-9)
Supplement: Supplementary file 1 — Figure S1. The structure of the category and effect measurement of CINV. The CR rates in the TRIPLE study were 59.1% (244/413 patients) for the GRA group and 65.7% (272/414 patients) for the PALO group. Regarding the case of CR or non-CR, we devised three categories according to the development of overall CINV as well as acute and delayed CINV (category 1, CR [0–120 h]; category 2, non-CR delayed; category 3, non-CR acute). (PPTX 83 kb) [file 40780_2018_128_MOESM1_ESM.pptx]

## Slide 1
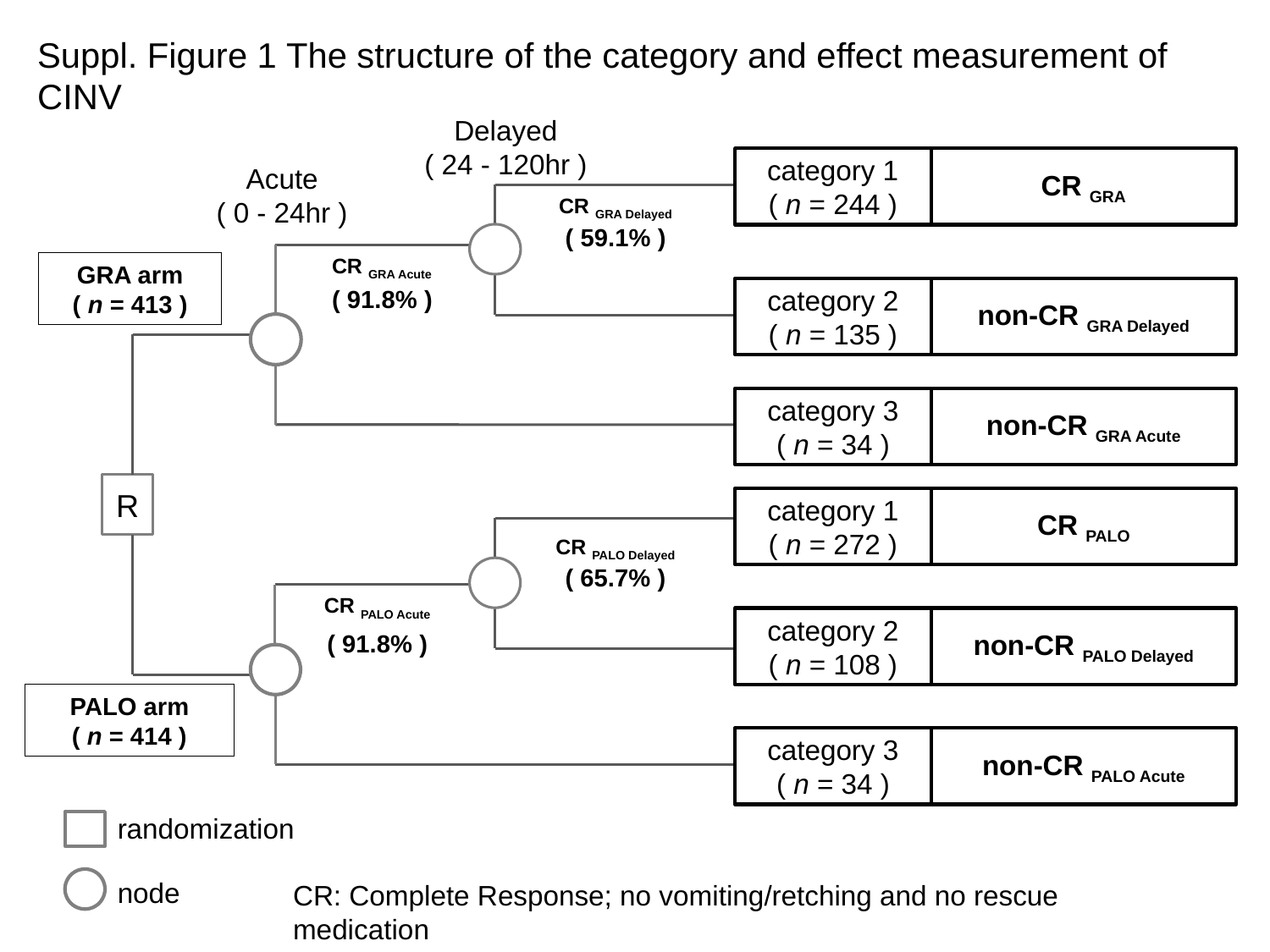

Suppl. Figure 1 The structure of the category and effect measurement of CINV
Delayed
( 24 - 120hr )
category 1
( n = 244 )
CR GRA
category 2
( n = 135 )
non-CR GRA Delayed
category 3
( n = 34 )
non-CR GRA Acute
category 1
( n = 272 )
CR PALO
category 2
( n = 108 )
non-CR PALO Delayed
category 3
( n = 34 )
non-CR PALO Acute
Acute
( 0 - 24hr )
R
CR GRA Delayed
( 59.1% )
CR GRA Acute
GRA arm
( n = 413 )
( 91.8% )
CR PALO Delayed
( 65.7% )
CR PALO Acute
( 91.8% )
PALO arm
( n = 414 )
randomization
node
CR: Complete Response; no vomiting/retching and no rescue　medication
